# Perinatal intimate partner violence in Quebec during the COVID-19 pandemic: victims’ help-seeking experiences and health and social care providers’ response

**DOI:** 10.1186/s12889-025-24736-3

**Published:** 2025-10-22

**Authors:** David Jean Simon, Geneviève Lessard, Sylvie Lévesque

**Affiliations:** 1 Centre de Recherches Appliquées et Interdisciplinaires sur les Violences intimes, familiales et structurelles (RAIV), School of Social Work and Criminology, Laval University, Quebec, Canada; 2 Department of Sexology, University of Quebec at Montreal, Montreal, Canada

**Keywords:** COVID-19 pandemic, Perinatal victims, Pandemic‑related barriers, Coping strategies, Resilience

## Abstract

**Introduction:**

Despite intimate partner violence (IPV) having increased significantly during the COVID-19 pandemic in Canada, it remains under-documented in the Quebec province. To partially fill this gap, this study sought to investigate IPV experienced by women in the perinatal period in Quebec and health and social care providers’ response in caring for both victims and their fetuses/children at the time of health restrictions.

**Methods:**

The study adopted a qualitative exploratory descriptive design and involved eleven perinatal victims and eleven health and social care providers (HSCPs). Semi-structured interviews were conducted using in-depth interview guides. Data was analyzed using thematic analysis.

**Results:**

The study showed the multifaceted dimensions of perinatal IPV during COVID-19. Spatial, psychological, physical, economic and sexual violence were reported by the perinatal victims. Further, we found that the type and magnitude of perinatal IPV varied greatly with the periods of restrictions. The study also indicated that perinatal victims faced spatial, institutional, cultural, geographical and perpetrator-related barriers to seeking help. For accessing formal and informal support resources, and escaping IPV, perinatal victims used diverse coping strategies. Additionally, we found that a wide range of barriers limited the ability of HSCPs to operate during the pandemic. However, to enable continued client engagement, they used several coping strategies such as remote working, hybrid IPV care (in-person and virtual services), close collaboration with other community organizations and pharmacies, food or gift card distributions; which reflects their ability to adapt and be creative to address challenges presented by the COVID-19 pandemic.

**Conclusion:**

This study shed light on the necessity to improve the provision of health and social services to better prevent IPV and meet the needs of perinatal victims and their fetuses/children.

**Supplementary Information:**

The online version contains supplementary material available at 10.1186/s12889-025-24736-3.

## Introduction

A novel coronavirus (COVID-19), discovered in the Chinese city of Wuhan at the end of 2019, spread rapidly around the world, infecting about 550 million people and leading to the death of more than 6 million in three years [1]. Due to its high rate of spreading and high mortality risk, the World Health Organization (WHO) declared COVID-19 a pandemic on March 11, 2020 [2]. In response to this health crisis, many governments imposed restrictive measures including Stay-at-Home order, hand hygiene, social distancing, wearing masks in public indoor settings, and closing schools and non-essential social services [3].

Although these preventive measures significantly reduced the spread of COVID-19 [4], they were accompanied by a rise in intimate partner violence (IPV) [5], defined as physical, sexual, psychological, or economic violence that occurs between former or current intimate partners [6]. Pre-pandemic, about one in three women worldwide, reported having experienced IPV at least once in their lifetime [7]. A recent study by UN Women conducted in 12 countries from April to September 2021 found that 45% of women (aged 18 and above) had been exposed directly or indirectly to at least one form of IPV since the COVID-19 pandemic [8], whereas pre-pandemic, IPV was below 40% in these selected countries [9]. A quantitative study by the Australian Institute of Criminology (AIC) involving 15,000 Australian women revealed an increase in IPV in the first three months of the pandemic : around 5% had been subjected to IPV, of which two-thirds indicated that the violence had escalated or started during the COVID-19 pandemic [10]. In Brazil the increase was between 40 and 50% following the introduction of mobility restrictions [11]. More specifically the city of Rio de Janeiro and the state of Parana saw respective increases of 50% and 15% in IPV reported to the police in the first week of lockdown [12]. Eleven days after the imposition of pandemic-related lockdowns (March 17–28, 2020), France recorded a 30% increase in IPV cases [13]. A systematic review and meta-analysis of 12 US studies showed that IPV incidents increased by 8.1% in the USA after COVID-19 lockdown mandates were imposed [14]. Although the above studies had shown how violence increased during the COVID-19 pandemic in several countries, a strict comparison between them would be biased due to their differences in methodology and timeframe.

In addition, calls to IPV helplines increased by 20% in several areas of the United Kingdom (UK), Spain, and France [15], 25% in Argentina, Germany, Mexico and the USA [13, 16], 30% in Cyprus, 33% in Singapore, more than 40% in Brazil, about 50% in Peru and Lebanon [17, 18], and 75% in Chile [19], around 2 weeks following the lockdown imposed by the governments of these countries in March 2020. Between March and April 2020, a 38% increase in the use of the 1800RESPECT (national helpline) was observed in Australia [20]. Moreover, in the seven days after stay-at-home policies, a 150% increase in visits to the Refuge (a domestic abuse organization) website was observed in the UK [21]. Likewise, ahead of March 30, 2020, searches for IPV help support increased by 75% on Google [22]. Note, however, that in the absence of specific studies on the types of help-seeking strategies used by victims to deal with IPV and their evolution during the pandemic, these estimates should be compared with caution. This unhoped-for worldwide situation has been labelled “Shadow pandemic” by the United Nations [8]. Based on existing literature, conditions like social isolation, financial insecurity (job loss and unemployment), poverty-related stress, more time spent at home with abusive partners (granting more freedom to perpetrators to exert their power/control), psychological distress, unease and fear, anxiety, depression and problematic substance use amplified the risk of IPV during the pandemic [13, 18].

IPV occurs across all social groups and stages of life. Even Women in the perinatal period were also profoundly affected during the pandemic. A recent systematic review found that perinatal IPV prevalence was 22% [23], whereas pre-pandemic, it ranged from 2 to 13.5% globally [24]. Other related studies indicated that perinatal IPV was 15.1% in Ethiopia [25], 18% in Wuhan (China) [26], 35% in Abbottabad (Pakistan) [27], and 35.2% in Tabriz (Iran) [28] at the time of the COVID-19 pandemic.

Importantly, health restrictions greatly affected social and health services for IPV, particularly at the beginning of the COVID-19 pandemic. While studies performed in USA [29], Finland [30], Australia [31], and Sweden [32] indicated that social and health services were reconfigured, as a result of physical distancing rules,—limiting their capacity to host and improvising to provide support for their clients (victims of IPV and others) —, the coping strategies adopted by health and social care providers (HSCPs) to enable continued client engagement remain under-addressed in the literature.

IPV is a major problem with a wide range of short- and long-term social and health consequences, including bodily injuries, gastrointestinal disorders, chronic pain, low self-esteem, depression, head trauma, suicidal thoughts, chronic mental illness, activity limitations, sexual dysfunction and sexually transmitted infections (STIs) [21]. Abused women in the perinatal period are at elevated risk for experiencing pregnancy complications, prenatal bleeding, premature labor, ruptured spleen, low birth weight, and miscarriage [3]. Moreover, fetuses/children exposed to perinatal IPV tend to be stunted, have low birth weights, and develop aggressive behaviors [33].

Canada’s situation was not all that different from the rest of the world during the pandemic. Statistics Canada have shown that 10% of women were extremely concerned about IPV in their homes since the implementation of social distancing and lockdown measures [34]. Besides, around 130 femicides were registered in 2020, compared with 89 in 2019 [35]. Also, calls to IPV helplines surged by more than 60% between September - December 2020, compared with the same period in 2019 [36]. In Alberta and Ontario provinces, calls increased by 20% [15]. Similarly, in the Quebec province, the number of femicides was 26 in 2021, as opposed to 23 in 2020 and 13 in 2019 [37]. Around 572 complaints of IPV were also recorded in March 2019 in the Quebec province, compared with 692 for the same period in 2021 [38]. Furthermore, the Quebec National Bureau of Statistics reported that, from the 6.6% pregnant Women sampled, 29.3% had experienced IPV during pregnancy in a survey carried out between 2020 and 2021 [39]. Equally, health and social agencies for IPV in Quebec revealed that the pandemic affected their working conditions and undermined good practice [40]. Despite the above findings and the harmful consequences of IPV for both women and fetuses/children, studies that shed light on this phenomenon in Canada at the time of COVID-19 are still lacking. Further, extant studies focused more on rising risks of IPV during the COVID-19 pandemic [41], and overlook the health and social care providers’ response, their experiences as well as those of victims including perinatal women – a sub-group who are often isolated [42] and facing even greater challenges in obtaining necessary and timely healthcare [43].

### Objectives

Perinatal IPV is a major public health concern and a violation of the Declaration of Human Rights [7]. To prevent this issue and better meet the needs of the victims at times of crisis such as the COVID-19 pandemic, comprehensive studies are crucial, and provide an important step towards achieving the Sustainable Development Goal (SDG) 5: “Achieve gender equality and empower all women and girls” [44]. Therefore, the current study aimed to contribute to the limited body of population-based research on perinatal IPV in Quebec (Canada). Specifically, our comprehensive study sought to: (i) document the barriers faced by perinatal victims in seeking help, (ii) explore the types of help-seeking strategies used by victims to deal with perinatal IPV, (iii) expose pressures confronted by HSCPs in responding to these challenges, and finally (iv) to explore the coping strategies adopted by HSCPs to identify victims and address perinatal IPV, including mechanisms implemented to ensure continuity of care during the COVID-19 pandemic.

## Materials and methods

### Operational definition of perinatal IPV adopted in the study

No consensus has yet been found about the definition of IPV in the perinatal period. It may vary from one study to another [41, 45]. In our study, perinatal IPV was defined as “violence or abuse that occurs during pregnancy and up to 2 years post-partum” [46]. IPV in the pre-pregnancy period has already been well documented in Quebec [47]. Thus, the results of this study will complement those conducted during the pre-pregnancy period.

### Study design

Our study used a qualitative exploratory descriptive design to facilitate flexible and in-depth discussions with the participants.

### Study population

Two (2) groups of respondents were targeted for this study: women who experienced perinatal IPV and HSCPs working in shelters and other community organizations providing services to women who experienced IPV.

### Data collection and procedures

An email detailing the study’s objectives, pre-defined criteria and research protocol was sent to health and social services administrators in Quebec, to pave the way for data collection with HSCPs. We also spoke to these administrators via Zoom/Teams to explain the procedures. The HSCPs were informed of the nature and procedures of the study by their administrators or team leaders. Further, posters were displayed in the health and social services break room and/or cafeteria to keep the HSCPs’ attention. Administrators and/or team leaders also reminded HSCPs of the importance of their participation in the study during weekly morning meetings.

To recruit women who experienced perinatal IPV, a hard-to-reach group, several strategies were developed, including: (i) soliciting help from HSCPs and managers of organisations providing services to women victims of IPV (shelters, perinatal services, and pharmacies); (ii) using social networks (Facebook page of the authors’ organization, Kijiji); (iii) sending recruitment emails to students and staff of Laval University (which was done by the information technology team of the University); (iv) and snowball recruitment, i.e. asking interviewed participants to refer other eligible ones.

All interested participants (women/HSCPs) contacted the research team using the available contact details. The date and time of the interview were agreed jointly by the first author and each interested participant. Interviews were conducted either by Zoom or face-to-face in a secure place mutually decided by the first author and the participants. The other two authors supervised the data collection process. A compensation of C$40 was received by the women interviewed to cover travel and childcare related expenses, as well as the time spent participating in the study. Overall, eleven (*n* = 11) women experienced perinatal IPV and eleven (*n* = 11) HSCPs were interviewed. We planned to stop recruitment after achieving thematic saturation, which occurs when no new themes were identified during interviews [48]. For the HSCPs group, saturation was reached after 11 interviews, meaning that no further information emerged in the interviews. By contrast, the recruitment of perinatal victims was difficult, and as a result, data saturation could not be complete for all themes. The sample size of 22 interviews (11 for each social group) was deemed sufficient for this study, as the data were rich and provided a wide range of information to address the research questions in depth. We do, however, recognize the small sample size as a limitation of the study.

Interviews with HSCPs lasted about 1 h, while those with the Women lasted between 1h30-2 h. HSCPs were recruited over the period June-November 2023, while the Women were recruited between January-August 2024. Starting with the HSCPs recruitment was a well-thought-out strategy, as their help was crucial in recruiting perinatal victims, a more difficult group to reach.

### Inclusion criteria

Inclusion was limited to HSCPs involved in the health and social services field and had cared for at least one perinatally abused woman during COVID-19 in Quebec. In addition, HSCPs were able to be interviewed in either French or English, the two official languages of Canada.

Women who experienced perinatal IPV were eligible if they were at least 18 years old at the time of the interview; had experienced perinatal IPV after March 2020, and were able to be interviewed in French or English.

### Data collection instruments

Data were collected through individual semi-structured interviews. An interview guide was developed by the research team for each specific social group. The HSCPs’ interview guide included questions about: (i) their perception of women who experienced perinatal IPV and its impact on their health as well as that of their fetuses/children; (ii) the obstacles faced by women who experienced perinatal IPV in seeking help; (iii) the barriers faced by HSCPs in providing care and (iv) strategies adopted by HSCPs to identify and care for victims and their fetuses/children during the pandemic. The women’s interview guide focused on perinatal IPV that they experienced during COVID-19; the impact of perinatal IPV on their lives and that of their babies/children; seeking help and obstacles; and needs of both women and babies/children to better cope with perinatal IPV. The women participants also filled out a form called “Help pathways”, enabling them to list the various resources with which they were in contact, in chronological order. Furthermore, both HSCPs and the women completed a socio-demographic questionnaire, which served to draw up their socio-demographic profile. Finally, we pilot-tested interview guides with two participants (a perinatal victim and a HSCP) and made the necessary adjustments to ensure high-quality research.

### Data analysis

All interviews recorded digitally were transcribed in verbatim form by the first author. To ensure the accuracy of the transcription, a double-check was carried out by the first author by listening to the audio recordings again and comparing them with what had already been transcribed. Further, thematic content analysis [49] was used to explore the women’s and HSCPs' experiences in-depth, and was facilitated using Atlas.ti software to code and organize the data. The first stage of the analysis involved generating codes after familiarisation with the verbatim. This process was guided by a coding frame with two sections: (i) a section on previous existing codes derived from qualitative studies on IPV experienced by women during COVID-19 [50] and (ii) an open-ended section of inductive codes derived from the study participants’ own experiences. The second stage of the analytical framework involved identifying the linkages between codes, themes and appropriate quotes from the participant interviews, and existing research. By the end of the process of analysis, these main themes were identified: changes in perinatal victims’ lifestyles; generation of new relational dynamics; changes in type and magnitude of perinatal IPV; barriers to help-seeking; challenges for practice; perinatal victims’ coping strategies; changes in service delivery approaches; help pathways toward ending violent relationships and safety planning; and post-separation monitoring.

### Ethical considerations

Our study was conducted in accordance with the Declaration of Helsinki. The research protocol received approval from the institutional review board of the Centres Intégrés Universitaires de Santé et de Services Sociaux (CIUSSS) de la Capitale-Nationale (Project # 2023–2853). Prior to enrolment in the study, each interested participant signed a consent form. The participants received the interview guide before the interview and were given the opportunity to ask questions and to seek clarification at any time throughout the interview, to avoid any influence. We were aware that some women might be uncomfortable talking about their experiences of IPV with a male interviewer. Therefore, before the interviews, we asked them for their preference regarding the gender of the interviewer. It was one way of potentially making more equal the power between researchers and participants. They were also informed that their participation was voluntary and that they could withdraw from the research study at any time without prejudice. In addition, to protect their anonymity, as well as that of the institutions they were affiliated with, pseudonyms were used in lieu of participants’ real names in report findings. All interviews were recorded on Zoom/computer, with the participants’ agreement, and transferred into the Laval University password secured drive of the first author. Access to the data is restricted to the research project team. Given the sensitive nature of the topic, it was vital to ensure the emotional safety of all participants. Their emotional reactions were carefully observed throughout the interview. If a participant felt upset during an interview, a break was offered. They were also asked if they wished to end and/or postpone the interview. None of the participants ended the interview. In addition, before concluding the interview, perinatal victims received a document containing information on access to health and social services.

## Results

The results section is structured as follows: first, we present the socio-demographic profiles of both perinatal victims and HSCPs. Secondly, each theme is presented and discussed in detail.

### Background characteristics of the study participants

At the time of data collection, their ages ranged from 27 to 58 years (Mean = 37.7, SD ± 10.4). Two (*n* = 2) had a college preparatory diploma (diplôme d’études collégiales in Quebec), five (*n* = 5) had a bachelor’s degree, while four (*n* = 4) had completed a master’s degree. Seven (*n* = 7) were social workers, one (*n* = 1) was a nurse, one (*n* = 1) was a gender sociologist, one (*n* = 1) was a psychologist, and the last one (*n* = 1) was a guidance counsellor. Additionally, they had an average of 8 years experience in their current role.

Perinatal victims ranged in age from 23 to 38 (Mean = 32.8, SD ± 5.0). Two (*n* = 2) were from rural areas. Further, six (*n* = 6) had a higher education level than a bachelor’s degree, one (*n* = 1) had completed a bachelor’s degree, two (*n* = 2) had a college preparatory diploma, and two (*n* = 2) had secondary education and below. Nine (*n* = 9) of the women were single mothers, whereas two (*n* = 2) were cohabiting with a partner. Seven (*n* = 7) were born in Canada while the other four (*n* = 4) were born abroad. Two (*n* = 2) had three children, five (*n* = 5) had two, and four (*n* = 4) had one child. Also, eight (*n* = 8) were shared-custody parents and three (*n* = 3) exercised sole custody of their children.

### Changes in perinatal victims’ lifestyles

The pandemic period was accompanied by considerable changes in people’s lifestyles. With the restrictive measures imposed by the Quebec government, most of the victims (*n* = 7) reported suffering from social isolation, notably during strict lockdowns.

« […] I was isolated from my friends and family. It was very hard for me because I couldn’t meet them. I no longer had those contacts, which are important to me. I suffered a lot, especially at the beginning of the pandemic. I was very nervous about the pandemic. » Celestine.

Economically, six (*n* = 6) encountered serious financial issues, of which five (*n* = 5) were due to pay cuts or job loss associated with the downturn in the activities of the company they worked for. Schools and day-care center closures also led some women to leave the job market, significantly reducing their financial autonomy. Clarah, a victim, said:

« I had 3 children when COVID-19 started. My youngest had just turned one. I didn’t work until June 2020. I was obliged to stay at home with the children, as the schools and day-care centers were closed […] I received CERB payments, but it wasn’t enough. » Clarah.

Additionally, some victims (*n* = 2) experienced severe fear about getting infected with COVID-19. Being pregnant, they were very worried about their unborn child, which impaired their ability to carry out their daily activities.

« I was pregnant during COVID […] In the media, it was said that pregnant women were very vulnerable to COVID. I was very afraid of being infected. I was very afraid for my baby. I read a lot about the effects of the virus on pregnant women and babies. I was so afraid of the effects of the virus […] I spent most of the day in bed doing nothing. I suffered from a lack of motivation. » Nanie.

Note, moreover, that most victims suffered from several issues simultaneously.

### Generation of new relational dynamics

COVID-19, with all its challenges, was not without repercussions on interpersonal behaviour. There was a generation of new relational dynamics and a rise in couple conflicts. Several victims (*n* = 3) shared how social isolation caused a lot of stress in their perpetrators, who suddenly became aggressive towards them.

« In the beginning, we were very close. The deterioration in our relationship was more due to the social isolation he experienced. He was very stressed because he couldn’t see his friends and family. More and more, he would reproach me for little things like a clothing that had fallen to the floor or if I moved his things. He blamed me for the food. He said it wasn’t good. The reproaches intensified. When I said to him, ‘Are you stressed? He was getting more nervous […] Every time I tried to talk to him, he was very aggressive. He isolated himself a lot in the bedroom. […] He was very depressed during the lockdown. » Rachelle.

Besides, other victims (*n* = 2) indicated that the financial stress faced by their abuser during COVID-19 was the main cause of their onset of couple conflicts. However, for over half the victims interviewed (*n* = 6), the effects of the pandemic were far from being the origin of their couple conflicts. IPV had occurred in some cases (2/6) when the perpetrators refused to accept the victims’ pregnancy, as Nanie narrated in the following way:

« When I got pregnant in February 2020, everything went to hell. He asked me to have an abortion, and I refused to do that. Doctors had already told me I had ovarian problems. When a woman has this kind of problem, it’s hard for her to get pregnant. I was never going to have an abortion. It broke up our relationship. » Nanie.

Further, two other victims (2/6) referred to their perpetrator’s excessive jealousy and substance addictions to explain their violent relationship.

« It was because of jealousy […] It was always jealousy. If he sent me a text message and I didn’t reply in 2 min, he imagined I was with a pilot. One day he told me that if I didn’t reply quickly, it was because I was *** a pilot. That was paranoia. I’m an air traffic controller […] » Sandy.

### Changes in type and magnitude of perinatal IPV

The COVID-19 pandemic brought significant changes in the dynamics of violence. Not only did it exacerbate new IPV and existing problems in many couples, but we also found that the type and magnitude of perinatal IPV varied greatly with the periods of restrictions.

#### O strict lockdown periods

Psychological violence, physical violence, and controlling behaviour were more intense during lockdown periods, as reported by most women exposed to these types of IPV. The more time passed, the more violent the perpetrators were, due to increased social isolation and negative effects of the lockdown on their mental health. Further, given that stay-at-home orders forced victims to remain in close proximity to their perpetrators, the perpetrators had more latitude to control behaviour and physically abuse them.

« We were always together in the same apartment, so he would hit me and pulled my hair. I was routinely physically abused. When I told him I was going to report him to the police, he confiscated my phone. He’d tell me that if I went outside, he’d report me to the police, because we were in strict confinement […] He also controlled me a lot. He wouldn’t let me out of his sight. It was hard to experience this during my pregnancy » Vanicia.

Financial violence was also more common during the lockdown period for some victims. Emeline and Paulette related that their perpetrators’ financial stability decreased at the start of the pandemic, forcing them to cover the largest proportion of family expenses.

« […] We had to pay for the mortgage. As his salary was cut during this period, he told me he was short of money and couldn’t contribute because he had to take care of his daughter (who wasn’t living with us), whom he had from another union. I had to dip into my savings to cover all the expenses. He told me he would reimburse me, but he never did. He had a lot of control over my expenses too. He would go into my online accounts to see how much I spent. He would transfer money to his daughter’s account via my account without asking me. » Emeline.

Another financial abuse victim (Steph) stated how social isolation exacerbated her perpetrator’s addiction to substances, and how he used her credit cards without her permission to buy substances.

#### O post-strict lockdown periods

Most sexual abuse victims reported increased incidence during the post-strict lockdown periods.

« He forced me to have sex with him once during confinement. I told him there’d be no next time because I’d lost my libido due to the intense violence and my pregnancy. At one point, we didn’t even sleep on the same bed […] When the restrictions were relaxed, the tension between us really decreased because he was less present in the house. As there was less tension between us, he took advantage of the situation to have sex with me. On several occasions, he would penetrate me during the night without my consent. When I didn’t agree, he said I was cheating on him with other men when he wasn’t home. » Celestine.

Meanwhile, one sexually abused victim did not observe significant change in the frequency of sexual assault across different periods of confinement. She even ignored that she was being sexually abused by her perpetrator, due to lack of knowledge and cultural values.

« I’m a Latin woman. In my culture it’s disrespectful to refuse sex to your husband. When I said no to him, he told me he was my husband, and I had to satisfy his desire. It was always like that in our relationship. I didn’t know it was rape; a social worker had told me I was being sexually abused. » Danise.

Regarding cyber-violence, it was only recorded in post-strict lockdown periods. Given that some outings were authorized, the perpetrators hacked into the victims’ phones to surreptitiously monitor their communications and location.

« He had installed my WhatsApp on his computer to access my text messages. He didn’t want me to tell other people about the situations I had experienced during the confinement. » Vanicia.

### Barriers to help-seeking

Despite that most victims were aware that their situation required emergency attention, they were faced with multiple challenges to accessing perinatal IPV services, which varied depending on the period of restrictions.

#### O strict lockdown periods

Four interviewed perinatal victims identified the constant presence of the perpetrators at home and the restrictions on meeting new contact and access to IPV services as their main barriers to help seeking, during this period. Similarly, daycare centers and school closures constituted obstacles in strict lockdown periods. In some cases, victims were caring for their children without assistance, which made it difficult for them to seek help. Rachelle said:

« We couldn’t visit our families and friends. Everything was closed. My youngest child (a baby) was at home, and I took care of him. My perpetrator didn’t help me. My days were very tiring. It wasn’t easy for me to seek help. » Rachelle.

Accessibility issues (long waiting times to contact and access services, lack of accommodation for perinatal women as well as saturation of shelters’ capacity), geographical location and rural conditions also posed significant challenges for victims (*n* = 2) seeking healthcare and support services.

« I lived in a rural area and had very young child. Getting around with them was difficult. We had a car, but my perpetrator used it all the time. Services were about an hour’s drive from where I lived. It was very far. » Clarah.

Two victims likewise shared that they were afraid of seeking help because their perpetrators used to snoop into their mobile phones and Facebook accounts during lockdown periods. Conversely, some victims (*n* = 3) did not seek any assistance during lockdown periods, for reasons not directly related to their perpetrators’ behaviour. One explained that she was not interested in the support remotely offered. She wanted to interact with services in-person because she felt too isolated. Another victim said she did not feel ready physically and emotionally to seek assistance, while the last one attributed her reluctance to seek help to cultural beliefs.

#### O post-strict lockdown periods

Although mobility and human interaction were less limited during post-strict lockdown periods, technological surveillance reduced some victims’ confidence in seeking formal assistance from support services.

« I felt I was being monitored everywhere and at all times, so even if I was a bit free, I couldn’t safely call social services and friends to tell them what I was experiencing […] On the way home once, he asked me what I was doing at the supermarket. I told him to stop spying on me. I felt suffocated in the relationship. » Sandy.

Equally, fear of increased IPV during post lockdown periods also created barriers which prevented victims (*n* = 2) for seeking support, as stated by Emeline:

« Things had calmed down a bit because he was spending some time outside. Although I wanted help, I didn’t seek it out because I didn’t want him to discover it and the violence to escalate again. » Emeline.

In addition, a victim expressed (Paulette) that she wanted to enjoy the easing of restrictions by spending time with her children and visiting friends/family, rather than seeking formal help.

### Challenges for practice

The pandemic not only posed problems for the perinatal victims, it also challenged HSCPs at different levels. Even though they were willing to help the victims, they were faced with all kinds of barriers and pressure to care for them properly.

#### O strict lockdown periods

Strict restrictions imposed by the government led health and social services to minimize physical interaction between HSCPs and victims by shifting to remote service delivery. Mikerline, a social worker, detailed:

« The first obstacle during strict confinement was the fact that we couldn’t meet face-to-face. The government had taken measures that we had to respect, despite our willingness to help. » Mikerline.

Several shelters were also forced to reduce available beds and divide their staff support numbers into small groups, working at different times. Claire, a HSCP, described the following:

« During periods of confinement, well, we were short-staffed to avoid the spread of COVID within our group. We had to split the team into small sub-teams to work at different times to avoid meeting each other. That way, if one team member is infected, at least it’s not the whole team, because otherwise we can’t offer services. » Claire.

Besides, some community social services operated much more by appointment, as Guerdite, a shelter staff explained:

« Activities were stopped and public spaces closed, so it was extremely difficult for us. But we said to ourselves, we’re going to stay in the field, we were there, we’ve always been there. The shelter was opened, I was in the workplace. We operated by appointment a lot. But if a victim came to seek help, we would receive her. » Guerdite.

Infrastructure challenges were also emphasized by HSCPs interviewed. The majority (7/11) revealed that the shelters in which they worked were not suitable for perinatal victims at the time of the announcement of strict confinement. Importantly, community social services were affected by the lockdown restrictions in often contradictory ways. If some were taxed with demand, others were lacking referrals. Cassandra, a HSCP, expressed her ideas in the following way:

« At the start of COVID, some shelters in Quebec were empty. Victims didn’t even know they existed before. But after the confinement, they received many requests because of the support of SOS violence conjugale (a national helpline). » Cassandra.

#### O post-strict lockdown periods

With an increase in calls registered during easing periods, health and social services managed to accommodate more perinatal victims. However, note that there was a mismatch between demand and capacity, creating pressure on staff, as the following quote illustrated:

« During easing periods, women were calling a lot. There were many more requests for accommodation, but we were obliged to reduce the number of women we could accommodate, so there were many more needs but fewer services. […] That put a lot of pressure on us. » Justine (HSCP).

Similarly, some community social services struggled to find volunteers during easing periods to operate their services. A shelter staff manager stated:

« […] After lockdown, we faced another obstacle. In fact, it was difficult for us to find volunteers to help care for the victims. Maybe people were afraid of being infected. » Mikerline.

### Perinatal victims’ coping strategies

To overcome the barriers to seeking formal or informal help faced during the COVID-19 period, perinatal victims used coping strategies which also reflected the periods of restrictions.

#### O strict lockdown periods

Paulette and Celestine described how they had sought psychological support from friends when their perpetrators were either out of home to go to the supermarket or asleep. Their friends advised them on the behaviours they could adopt to reduce the tension in their relationship and comforted them.

In the same vein, Rachelle revealed how she sent text messages to her psychologist when her perpetrator was playing video games or going to the bathroom.

« He liked to play video games and when he did, he put on his headset. As for me, I contacted my psychologist to find out what I could do in the situation I was in. I also sent messages to my psychologist when he also went to the bathroom. Then I deleted the messages. […] She’s been my psychologist since 2018. » Rachelle.

Sandy (a victim of cyberviolence) indicated likewise that she regularly used a work colleague’s phone on her lunch breaks to seek support.

« I worked during periods of restriction. During my lunch breaks, I used a colleague’s phone at work to call for help. I called the SOS violence conjugale helpline. I was referred to a shelter […] » Sandy.

#### O post-strict lockdown periods

As lockdown restrictions eased, more perinatal victims used coping strategies to seek formal or key informal support. Five (*n* = 5) shared how they had taken advantage of their perpetrators’ absence at home to contact or talk to close friends/family members and health and social service providers.

« During the lockdown, I hadn’t called because I wanted to meet in person with a professional. When he wasn’t home, I called friends. Also, I went to see some social workers at a shelter near my home who helped me. » Steph.

Furthermore, a victim mentioned that she had returned to work during the easing period and was using her break time to contact friends and health and social service providers.

« […] I was back at work during periods of partial confinement. When I was on break, I contacted friends and called social services for support. » Clarah.

Four other victims (*n* = 4) reported that they had frequently discussed their situation with family and friends when they visited them or were away from home but had not contacted health and social service providers. In Danise’s case, she did not seek help even during the easing period because her perpetrator manipulated her:

« Even if he wasn’t at home, I couldn’t call the health and social services provider, I couldn’t talk about my situation with my friends and family. He manipulated me, telling me that if I told people about it, it would affect the family’s image. » Danise.

### Changes in service delivery approaches

Within these essential services, health and social service providers exposed themselves and their loved ones to the risk of infection as they continued to effectively respond to need with varied methods. Indeed, to provide support for perinatal victims and handle increased responsibilities during the pandemic, adaptive measures had been developed.

#### O strict lockdown periods

In response to the restrictive conditions imposed by the quarantine periods, some health and social services intensified their social media presence and set up safety hotlines to increase awareness of IPV and encourage victims to break their silence and seek help.

« […] We stepped up our presence on our Facebook page to raise awareness and encourage victims to seek help. Our website wasn’t what it is today, but we had put up a message that we were open. We used social media a lot […] After that, we realized that our volume of calls for help had increased. » Guerdite.

Morgane, another HSCP, described how she organized virtual meetings via Facebook/Instagram apps, to contact and assist victims.

« I used to meet victims virtually (via Zoom) because we couldn’t see anyone face-to-face […] We (shelter staff) really started doing that with the outbreak of the pandemic arrived when everything was closed. » Morgane.

Several HSCPs (*n* = 3) recognized that virtual meetings were very risky in the presence of perpetrators. To ensure victims' safety, they adopted other strategies, such as secret hand signals, confidential codes to be informed by victims of a possible risk.

A protocol of cooperation had also been implemented by pharmacies and community social services to identify victims and refer them to the appropriate support services.

« There was a feeling of powerlessness at the start of the pandemic. We went to the pharmacies to get help. We left our [name of organization] leaflet in pharmacies. It was to make victims aware that if they and their children needed help, they could call the shelter. » Claire.

Besides, some health and social service providers went door-to-door in neighborhoods close to their sites to encourage neighbors to report IPV.

#### O post-strict lockdown periods

Collective kitchens, discussion circles, food or gift card distributions were adopted as additional coping strategies by many agencies’ social services during easing periods. They organized these events to identify, meet, and assist the victims, as well as to raise public awareness of perinatal IPV, as four (*n* = 4) HSCPs stated:

« We organized food distributions at the Center. During these distributions, we raised awareness and [took] the opportunity to talk to the women about their family environment. If a woman said something to us, or we saw her arrive with bruises, we would take the time to talk to her and refer her to the appropriate services. » Cassandra.

Notwithstanding these very real challenges, the pandemic also clearly created opportunities for changes to service provision across health and social service providers.

### Help pathways toward ending violent relationships and safety planning

**Fig. 1:**
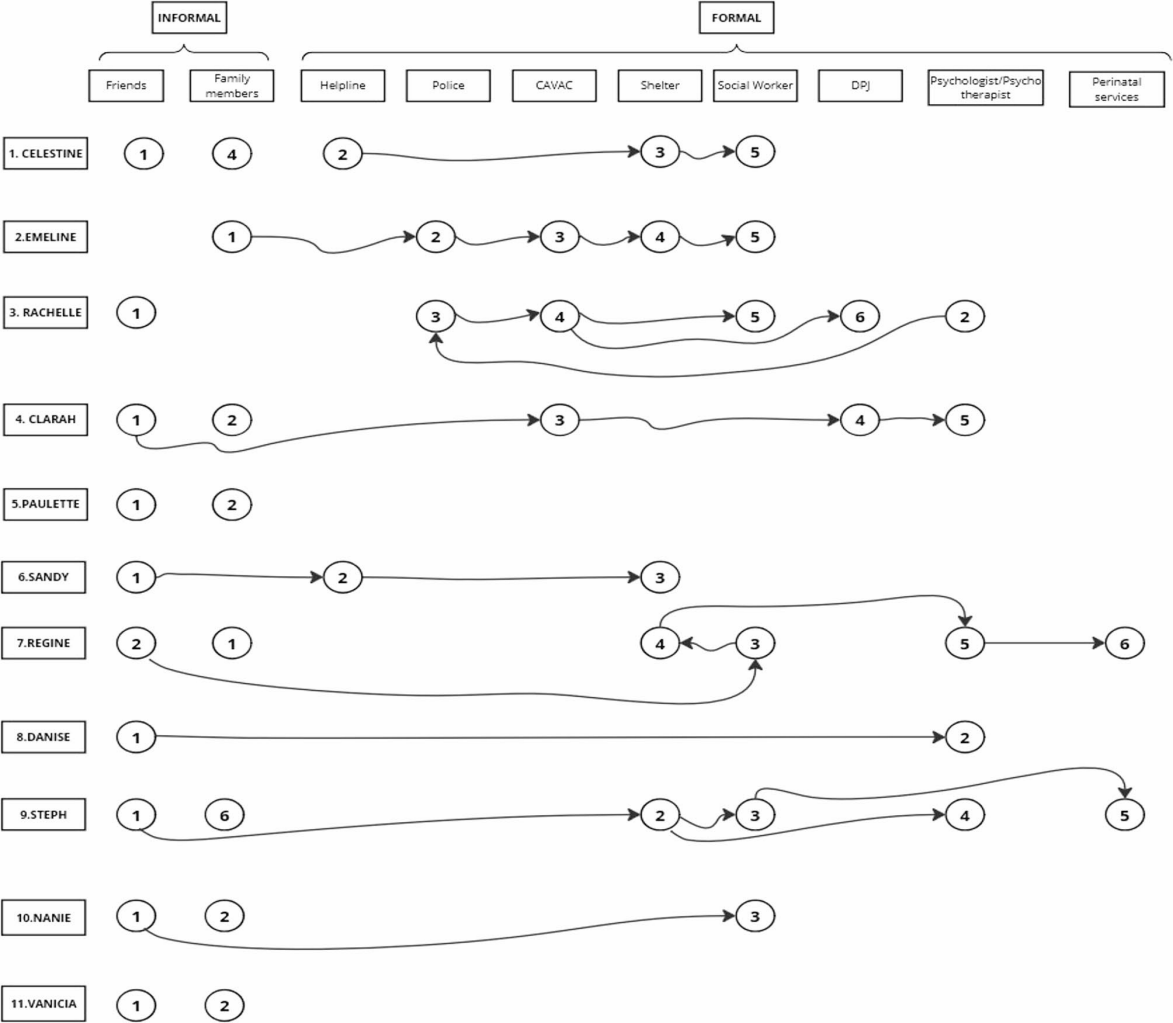
Help pathway of each perinatal victim. Abbreviations: CAVAC (Crime victims assistance centres); DPJ (Youth protection office in Quebec) READ: In Emeline's case, she shared her experiences of perinatal IPV with family members first, who then referred her to the police, who in turn referred her to a crime victims assistance centre. In addition, CAVAC put her in contact with a shelter, which in turn put her in contact with a social worker. In Paulette's case, she shared her situation first with friends and then with family members. They advised her to go to social services, but she did not follow-up

Consideration of perinatal victims’ help-seeking strategies provides insight into other decision-making processes. In Fig. 1 below, which illustrates the various resources with which they were in contact (in chronological order), it can be observed that family members and friends were the first to be informed about the perinatal IPV endured by most (*n* = 9) perinatal victims.

« I told my colleague (a friend) about my situation first. I didn’t tell her everything, but she quickly understood what I was experiencing and opened my eyes by telling me that it wasn’t right what my perpetrator was doing to me. » Sandy.

Informal help-seeking strategies helped victims to deal with perinatal IPV and provided important pathways for accessing formal resources that referred them to other formal sources of care. As in Regine’s case, her mother, with whom she first discussed her situation, advised her to go and see a social worker, who referred her to a shelter.

Importantly, contact with health and social service agencies led to perinatal victims regaining their self-confidence gradually, ending IPV and terminating abusive relationships.

« A friend of mine had told me about a shelter not too far from where I lived. They (professionals in the shelter) helped me leave the apartment where I was living with my perpetrator, but I didn’t want to be moved into the shelter with a pregnancy. I stayed with a friend until I found other accommodation. » Steph.

Note, nevertheless, that while majority of the victims (*n* = 7) did not wait long to leave their abusive relationships after being in contact with formal services, for others the process was much longer (more than 1 year) due to the fear of the moral judgment of their relatives, the fear of the consequences of the separation on their children or the fact that they had just given birth.

« […] Friends and social services proposed their help. But it took me a long time to accept that children can’t grow up in this situation. I was afraid that separating from my perpetrator would have repercussions such as stigmatizing the children. It took me a long time to separate from him. » Paulette.

### Post-separation monitoring

Community social services provided ongoing support for both perinatal victims and their children, even after they had been separated from their perpetrators. In addition to referring them to other resources, they accompanied them with other procedures and called them to check on their progress.

« After my separation from my perpetrator, a crime victim assistance center (CAVAC in Quebec) provided me with a psychologist. It helped me with everything I needed to do, either on a legal level or for advice. » Clarah

Individual and group meetings via zoom or face-to-face were organized regularly by certain organizations to keep in touch and follow up victims.

## Discussion

Our study investigated victims’ experiences in the Quebec province during COVID-19, as well as those of HSCPs who maintained a front-line practice throughout the pandemic; providing care for perinatal victims. To do so, a qualitative approach was adopted, by conducting semi-structured interviews with eleven (*n* = 11) perinatal victims and eleven (*n* = 11) HSCPs.

The results showed that the COVID-19 pandemic profoundly altered the lives of most perinatal victims interviewed, struggling from a loss with interpersonal ties. The extended periods of time spent in lockdown without formal and informal support negatively affected their mental health, creating heightened levels of stress and anxiety. Aligning with existing pre-pandemic research that has demonstrated a strong association between social isolation and psychological distress after a disaster or public health emergency [51], our finding supports a Durkheimian perspective that argues that the lack of social connectedness and companionship outside the home might have short- and long-term deleterious effects on individuals’ mental health and well-being [52].

Our study revealed that the COVID-19 pandemic has significantly influenced IPV experiences. Perpetrators’ loss of wages and social isolation contributed significantly to the occurrence of IPV in the relationships of the perinatal victims interviewed. Congruent with previous research [53], this finding confirms that these stressors were catalysts for IPV during COVID-19. They provided fertile ground for escalating IPV. Specifically in the case of social isolation, Stets [54] emphasizes that it can produce weakened social control, linked to the heightened risk of IPV. Substance abuse during COVID-19 was another critical factor associated with the onset of IPV, which corroborates prior studies [55]. The social and psychological distress during the COVID pandemic, along with the existing mental disorders in some perpetrators with substance abuse related disorders, have had a detrimental synergistic effect, increasing addiction rates and risks of overdose [56]. For several authors [57], substance abuse can impair judgment or cognition, and often lead to trust issues, communication breakdowns and emotional distress in relationships; making individuals more prone to perpetrating or becoming victims of IPV. Nevertheless, other researchers [58] have pointed out that many perpetrators use drinking or substance misuse as an excuse for their actions.

Although the pandemic served as a catalyst for IPV, generated a new cohort of perinatal victims, and exacerbated new perinatal IPV and existing problems in many relationships, our results demonstrated that all cases of perinatal IPV were not directly associated with pandemic-related stressors, as some media and numerous studies had reported [59]. This finding underlines the importance of conducting further research with survivors, to get much more nuanced and comprehensive evidence on IPV during the COVID-19 pandemic.

Another interesting point from our study is the changes in type and magnitude of perinatal IPV. Psychological violence was more prevalent during the pandemic, and for those who experienced coercive control, it was more intense during lockdown periods. This is consistent with a recent study in Portugal [50]. Arguably, stay-at-home orders allowed more freedom to several perpetrators to exert their power. Being permanently confined with the perinatal victims, they were able to intensify levels of surveillance and control, and “weaponized” the COVID-19 situation to abuse the victims without physically harming them [21]. Public and media narratives about IPV during the COVID-19 period were largely reduced to physical violence. Thus, our study suggests how the nature of non-physical forms of abuse may have evolved at the time of the pandemic.

In addition, various social-ecological factors were identified as barriers to help-seeking in this study. The prolonged time at home with perpetrators during strict lockdown periods restricted some victims’ space for action and further cut them off from all external support. This observation coincides with evidence from France [60]. These perinatal victims felt trapped within their own home, which was supposed to be a safe place for them and the children, with their perpetrators. In such a context, the home could be more dangerous for some perinatal victims than the COVID-19 pandemic itself. Bulteau et al. [61] described this situation (i.e. the way the perpetrators occupied the house, an unsafe space, to control the victims’ ever-changing movements) as “spatial violence”, rendering victims “unseen” to those outside the home and hindering their ability to seek help. Our results also showed that the “spatial violence” even extended beyond the home during post-strict lockdown periods, through spyware and surveillance technology used by perpetrators. Not only do they facilitate coercive and controlling behaviours (known as digital coercive control), but they also provide a way for perpetrators to stalk victims and be omnipresent in their lives [62]. Stark asserts that stalking is the most prevalent form of surveillance used in coercive control [63]. The use of these surveillance tools by perpetrators is a real threat to privacy. It represents a great danger to victims, who often use technological devices to seek help and ensure their safety and their children’s, and may further lead to other forms of IPV [64].

The presence of children at home was a relevant barrier to seeking help for other perinatal victims during quarantines, which is supported by another study [65]. The inequality in the distribution of family and domestic tasks intensified their isolation, reduced options and availability of help seeking efforts. Cultural beliefs about family and gender roles have also been found to be a relevant barrier affecting the help-seeking process for a Latin immigrant perinatal victim. This concurs with findings from past studies [50]. In many Latin cultures, women are expected to preserve the family from shame and promote family harmony. Respect for these cultural values is often used by perpetrators as a resource to silence victims [66]. Importantly, fear of increased IPV during post lockdown periods created barriers for few perinatal victims. In agreement with a study by Sabri et al. [67], this result illustrates that these perinatal victims had limited knowledge about the dynamics of IPV.

In addition to cultural beliefs, institutional and structural factors including reduction in shelter capacity to comply with isolation protocols, lack of accommodation for perinatal victims, and longer delayed response times presented a barrier to formal help seeking during lockdown periods. This evidence is in line with studies in Portugal [50]. According to Lapierre and colleagues [47], institutional and structural barriers can be seen as a form of “revictimization” for women and children. Besides being abused by the perpetrators, perinatal victims experienced institutional/structural violence that could reinforce their feelings of helplessness and preclude them from effecting any kind of escape from the relationship. Similarly, geographical barriers have also been identified in our study, which is in concordance with findings in the United States during COVID-19 [68]. People from rural areas in Quebec are under-resourced in IPV services than their urban counterparts. Achieving equity in access to social services and healthcare must be a priority for the Quebec government in addressing the geographical disparities.

In light of the above, the substantial decrease in requests for assistance from IPV victims to social agencies during lockdown periods in Quebec should not be interpreted as a reduction in IPV cases. Rather, it indicates that many victims had been confronted with several types of barriers that silenced them.

Further, our current study found that a wide range of barriers (infrastructure challenges, remote service delivery, reduction in support staff) limited the performance of the HSCPs during the pandemic. An earlier study in Ireland has reported similar findings [69]. Both perinatal victims and HSCPs had to deal with IPV incidents in a context that can also be characterized as traumatic, as Holt et al. [69] pointed out. Thus, perinatal victims and HSCPs were suffering the same collective trauma of the COVID-19 pandemic. The “shared trauma” or “shared reality” can lead some HSCPs undergoing post-traumatic stress to unconsciously violate the professional boundaries between caregivers and care receivers, and increase their self-disclosure to victims [70]. The “shared trauma” may raise ethical issues, as it can have a negative impact on the efficiency of service delivery. In any situation, HSCPs must recognize the sense of integrity that is essential to their profession. They must always make moral sense of the work, guided by professional ethics [70].

To overcome pandemic‑related barriers that prevented access to formal and informal support, the interviews revealed that most perinatal victims used coping strategies when perpetrators were away or busy, which included contacting friends, family members and help services, using friends’ phones to call social agencies, and visiting friends and family members during easing periods to discuss their situation with them. In line with previous findings in the pandemic context [71], this result demonstrates the capacity of these perinatal victims, - aware of their vulnerable situation and its potential impact on their health and that of their fetuses/children - to adapt to the dynamics of IPV and the health restrictions in seeking help. According to Trigueiro et al. [72], the use of these different strategies by perinatal victims to overcome multi-faceted barriers is the beginning of the resilience process. This result also underscores the importance of friends and family networks in seeking-help. In sharing their personal stories of IPV with their immediate social network, perinatal victims engaged in the process of overcoming the negative outcomes associated with victimization [73]. Friends and family members helped perinatal victims to deal with IPV and provided important pathways for accessing other sources of care and reporting IPV incidents. They must be considered as a complement to health and social agencies, supporting the Pescosolido’s Social Organization Strategy perspective [74], which conceives help-seeking as a set of overlapping decisions that may lead victims to turn to a wide range of informal and formal services. In summary, social support from friends and family networks facilitate HSCPs interventions and further participate in the development of resilience in women victims [74], a key part of the recovery process.

Equally, the study indicated that several coping strategies were adopted by social agencies such as remote working, hybrid IPV care, close collaboration with other community organizations and pharmacies, food or gift card distributions, to enable continued client engagement. Similar findings were reported in other settings [31]. The implementation of these new emergency measures by the health and social service agencies reflects their ability to adapt and be creative to address the challenges presented by COVID-19. The different coping strategies used by the health and social agencies illustrate the challenges that the COVID-19 pandemic and changed family dynamics placed on victims’ access to safe spaces [69], but also the relevance of strengthening community collaborations to address victims’ needs and safety during turbulent times. A strength of the collaborative approach is that it enables perinatal victims to access timely help to prevent further IPV and can provide optimal care for them and their fetuses/children. In other words, collaborative approaches in health and social service agencies can help to convince victims that IPV is not a private concern, and that they are not alone.

Importantly, the findings showed how technology was a valuable aid in identifying and caring for perinatal victims during the health crisis. However, while researchers asserted that remote technology has opened up creative opportunities for emergency adaptive changes to health and social work practices, they reported on the ethical challenges posed by its use, given the loss of face-to-face interactions which facilitate clearer disclosures and the difficulties of building trust fully and observing victims’ non-verbal behaviours [31].

Finally, different care arrangements have been set up by the health and social service agencies to ensure continuity of care for perinatal victims and their fetuses/children. This demonstrates the practical intelligence of HSCPs who recognize that “Care” is a process that makes possible the development and continuity of the process of leaving abusive relationships and of resilience in perinatal victims.

### Strengths and limitations of the study

The main strength of this study is the fact that it involved interviewing both perinatal victims and HSCPs. Note that the HSCPs are often overlooked in IPV studies in Quebec, whereas they hold relevant information on the topic as they deal with the victims. In this sense, information gathered in the interviews with the HSCPs complemented that of the perinatal victims. Moreover, the qualitative nature of the study provides in-depth information for future intervention programmes and public health emergencies. Nevertheless, this study is not free of limitations. Given IPV is a sensitive topic, perinatal victims may withhold certain information, which could influence the study findings. Although during the recruitment process, we sought to reach data saturation and have a variety of profiles of perinatal victims, people from disadvantaged socio-economic backgrounds and in precarious living conditions (e.g., immigrant women, women in rural areas) are underrepresented and people with higher education level are overrepresented. This overrepresentation and the non-saturation of data likely influenced the findings of this study. It could greatly limit our knowledge of the experiences of women with low educational levels or who were living in precarious conditions prior to COVID-19, such as refugees and homeless women. Our sample of perinatal victims interviewed is not representative of the population of perinatal victims in Quebec. Another inherent bias is the use of social media and the sending of emails to students and staff of Laval University to recruit perinatal victims. Women without social media accounts did not have a high likelihood of being recruited. This would generate a sample that is unbalanced in selected socio-demographic characteristics. As the recruitment of participants was on a voluntary basis, selection bias could also not be avoided.

## Conclusion

To the best of our knowledge, this is one of the few studies conducted in Quebec on the experiences of perinatal victims in seeking help, and of HSCPs in caring for perinatal victims during the COVID-19 pandemic. Methodologically, our study differs from others performed in Quebec in that it is the only one to include both perinatal victims and HSCPs. Besides, it is the only study to examine the help pathway of each perinatal victim.

Although the COVID-19 pandemic has had a negative impact on health and social service agencies, it has also given their managers an opportunity to think about new measures to improve them. The Quebec government and health and social service agencies must learn from the critical issues that emerged during the COVID-19 pandemic in helping perinatal victims and their fetuses/children, to develop effective strategies that can also be adapted to other emergency situations. Also, social service agencies need to capitalize on their experiences during COVID-19 and provide more training for HSCPs. This will increase their creative capacity and enable them to adapt swiftly to new public health crises.

Considering the foregoing results, adequate resources should be allocated to health and social service agencies, particularly those in rural settings, to increase their capacity, both in terms of staff and equipment, to meet a potential increase in needs, while accounting for isolation requirements. Community education should be intensified about the dynamics of IPV, - by posting videos on social media, distributing informative leaflets and handouts, etc. -, which would help more women to anticipate violent behavior from partners and avoid the worst.

The collaborative approach adopted by HSCPs was of paramount importance in addressing perinatal IPV during the COVID-19 pandemic. Although the Quebec government’s IPV action plan (2018–2023) obliged workplaces to support victims and ensure their safety, more local stakeholders should be invited to get involved in this relevant cause, which will make victims feel that they are not “alone” and that the fight against IPV is everyone’s concern.

## Supplementary Information

Supplementary Material 1.

## Data Availability

The data analysed in the current study are not publicly available to ensure the participants’ confidentiality, but may be obtained from the corresponding author on reasonable request.
