# Supplementary material for: Perinatal intimate partner violence in Quebec during the COVID-19 pandemic: victims’ help-seeking experiences and health and social care providers’ response
Source: BMC Public Health. 2025 Oct 22;25:3569. doi: 10.1186/s12889-025-24736-3 (PMC12542276; doi:10.1186/s12889-025-24736-3)
Supplement: Supplementary file 1 — Supplementary Material 1. [file 12889_2025_24736_MOESM1_ESM.pdf]

Supplemental materials

The interview guide for perinatal victims can be found on the following page.

Titre du projet : La santé des femmes victimes de violences conjugales en période périnatale et celle de leurs enfants : un enjeu de taille dans la crise sanitaire de la Covid-19 au Québec

Recherche postdoctorale-École de travail social et de criminologie

GUIDE D'ENTREVUE DES FEMMES

Script d'introduction

Bonjour,

Nous vous remercions d'avoir accepté de nous rencontrer aujourd'hui. Vous avez accepté de contribuer à notre projet de recherche à titre de participante. L'entretien individuel semi-directif dont nous nous sommes convenus a pour objectifs de/d' :

- *Comprendre le vécu de la violence conjugale en période périnatale (VCPP) durant la crise sanitaire*
- *Comprendre les impacts perçus de la VCPP sur votre santé (physique et mentale), ainsi que celle de vos enfants*
- *identifier les obstacles auxquels vous aviez fait face en ce qui a trait à la recherche d'aide*
- *explorer les stratégies adoptées par les professionnels de santé pour vous repérer et intervenir sur la VCPP*
- *Dresser un portrait de vos besoins et entendre vos recommandations*

Nous allons revenir ensemble sur le FIC, afin de nous assurer que tout est clair et que vous consentez à participer de manière libre.

À la fin de l'entrevue, vous aurez à remplir un questionnaire sociodémographique. Par ailleurs, si vous en éprouvez le besoin, nous prendrons le temps de discuter et nous vous présenterons la liste des ressources à consulter au besoin.

Tout au long de l'entretien, vous êtes libre de nous interrompre, de nous poser des questions ou de faire une pause ou de ne pas répondre à certaines questions si vous ne le désirez pas.

Violences subies par les femmes durant la crise sanitaire

1. Comment aviez-vous vécu la période pandémique ?
   a. Quels sont les défis auxquels vous aviez fait face ? (Ex : situation d'emploi, ressources financières/matérielles, isolement…) ?
   b. Quels sont les défis auxquels votre partenaire avait fait face ?
   c. Comment décrivez-vous votre relation avec votre partenaire avant la pandémie ? Parlez-nous de l'évolution du comportement de votre partenaire et de votre relation durant la crise sanitaire ?
2. Pourriez-vous nous décrire les violences que vous aviez subies de la part de votre partenaire ? (forme-s, contexte, exemples d'événements, entre autres)
   a. durant les périodes de confinement strict ?
   b. durant les périodes de ralentissement de la propagation (certaines sorties sont autorisées sans justificatif, ex : faire du sport) ?
   c. durant les périodes de dé-confinement partiel/total ?

   Relance : Si la répondante parle uniquement d'un certain type de violences à demander : auriez-vous vécu d'autres types de violences (psychologiques, financières, sexuelles)?
3. Pendant cette période, pourriez-vous nous expliquer comment votre partenaire s'est comporté avec votre/vos enfant (s) ?

    a. Aviez-vous constaté un quelconque changement dans son comportement à l'égard de vos enfants ?

Impacts de ces violences sur les mères et les enfants

4. Pourriez-vous nous parler des impacts de ces violences sur vous ?
    a. sur votre situation financière ?
    b. sur votre santé physique ?
    c. psychologique et psychique ?
    d. sur votre rôle de mère ?
    e. sur la relation mère-enfant ?
5. Pourriez-vous nous parler des impacts de ces violences sur vos enfants ?
    a. sur leur santé physique ?
    b. sur leur santé psychologique/émotionnelle ?

Recherche d'aides et obstacles

6. Vous êtes-vous confiée au sujet de ces violences ?
   Si oui :
    a. À qui vous vous êtes confiée en premier au sujet de ces violences ?
    b. Comment cela s'est-il déroulé ?
    c. Vous a-t-il dirigé vers des ressources d'aide ?

   Si non : pourquoi ?
7. Pourriez-vous m'en parler des demandes d'aides que vous aviez faites ? (à présenter la fiche de ressources-parcours d'aides à la répondante, **voir Annexe 1**). Exemple ressources (ami.e.s/famille, Justice, Intervenants services sociaux et de la santé,…)
8. Comment ça s'est passé ? Qu'est-ce que vous avez apprécié et moins apprécié de chacune des ressources que vous aviez indiquées précédemment ?
9. Pourriez-vous nous parler aussi des obstacles rencontrés en ce qui concerne la recherche d'aide auprès de chacune de ces ressources ?

Besoins des mères et des enfants

10. De quoi auriez-vous besoin pour mieux faire à ces violences que vous aviez vécues ainsi que vos enfants ?
    a. en lien avec les services sanitaires ?
    b. en lien avec les intervenants en services sociaux et sanitaires ?
    c. en lien avec les services juridiques comme la police ou les tribunaux ?

11. Pour finir, voudriez-vous parler d'autres aspects importants non abordés ou auriez-vous des suggestions/recommandations ?
    a. en lien avec la violence
    b. en lien avec vos besoins ainsi que ceux de vos enfants
    c. en lien avec l'amélioration des services d'aide et de santé relatifs aux femmes victimes de VCPP.

Script de conclusion

C'est la fin de l'entrevue et nous vous remercions pour votre temps et votre témoignage.

Comme nous vous l'avions dit tout au début de notre rencontre, nous vous prions de bien vouloir remplir ce petit questionnaire concernant vos caractéristiques sociodémographiques (Remplissage : 5 mn).

Remise du dédommagement.

Présentation de la brochure de ressources et offre d'un temps de discussion / soutien au besoin.

Remerciements et salutations.
